# Increasing Alternative Promoter Repertories Is Positively Associated with Differential Expression and Disease Susceptibility

**DOI:** 10.1371/journal.pone.0009482

**Published:** 2010-03-01

**Authors:** Song Liu

**Affiliations:** 1 Department of Biostatistics, Roswell Park Cancer Institute, Buffalo, New York, United States of America; 2 Department of Biostatistics, School of Public Health and Health Professions, The State University of New York at Buffalo, Buffalo, New York, United States of America; 3 New York State Center of Excellence in Bioinformatics & Life Science, The State University of New York at Buffalo, Buffalo, New York, United States of America; University of Pennsylvania, United States of America

## Abstract

**Background:**

Alternative Promoter (AP) usages have been shown to enable diversified transcriptional regulation of individual gene in a context-specific (e.g., pathway, cell lineage, tissue type, and development stage *et. ac.*) way. Aberrant uses of APs have been directly linked to mechanism of certain human diseases. However, whether or not there exists a general link between a gene's AP repertoire and its expression diversity is currently unknown. The general relation between a gene's AP repertoire and its disease susceptibility also remains largely unexplored.

**Methodology/Principal Findings:**

Based on the differential expression ratio inferred from all human microarray data in NCBI GEO and the list of disease genes curated in public repositories, we systemically analyzed the general relation of AP repertoire with expression diversity and disease susceptibility. We found that genes with APs are more likely to be differentially expressed and/or disease associated than those with Single Promoter (SP), and genes with more APs are more likely differentially expressed and disease susceptible than those with less APs. Further analysis showed that genes with increased number of APs tend to have increased length in all aspects of gene structure including 3′ UTR, be associated with increased duplicability, and have increased connectivity in protein-protein interaction network.

**Conclusions:**

Our genome-wide analysis provided evidences that increasing alternative promoter repertories is positively associated with differential expression and disease susceptibility.

## Introduction

Promoter is the region of DNA consisting of transcriptional regulatory elements required for transcription initiation. Alternative Promoter (AP) usage refers to the control of alternative transcriptional start within a single gene locus by using alternative promoter. AP usage has been observed for many individually characterized genes [1], [2] and recent genomic studies have found that approximately 50% of human genes have at least one AP [3], [4]. The wide-spread AP usage indicates it might play a critical role in shaping human genome and transcriptome [1], [2], [5], [6].

As AP consists of different modules of *cis*-regulatory elements [7], [8], AP usage has long been explored for the regulation of expression diversity of individual metazoan gene [9]. For example, by selectively using one promoter active in parotid gland and the other active in liver, mammal *α-amylase* gene shows a more than 100-fold difference of expression level in these two tissues [10]. The number of individually characterized genes with AP driving context-specific (e.g., pathway, cell line, tissue type, development stage, species *et. ac*.) manner of differential expression has accumulated during the past two decades [1], [2], [5], [6], [11], [12]. This thus raises an interesting question: are genes with AP more likely to be differentially expressed than genes with Single Promoter (SP)? Furthermore, among genes with AP, are genes with more AP more likely to be differentially expressed?

There is also growing evidence that AP usage is linked to disease through aberrant promoter choice and/or genetic defects affecting the functional *cis*-regulatory element [2], [9]. For example, the upstream promoter of *MYC*, dominant negative in normal tissue, is aberrantly activated in Burkitt's lymphoma cells due to aberrant translocation of *MYC* gene locus [13]. A recent survey of mammalian AP showed that the group of putative human cancer related genes (∼2,800) on average have 2 promoters compared with an average 1.5 promoters among the other human genes [2]. However, cancer related genes can be classified into passenger and driver, with the later playing a critically causal instead of passive role in tumor formation and progression [14], [15]. It remains unclear whether there is a general link between a gene's AP repertoire and the likelihood of being cancer driving genes. Furthermore, it remains unclear whether or not there is a positive relationship between the increasing promoter repertoire and the likelihood of being associated with general human diseases.

## Results

### AP Genes Are More Likely to Be Differentially Expressed

**Figure 1:**
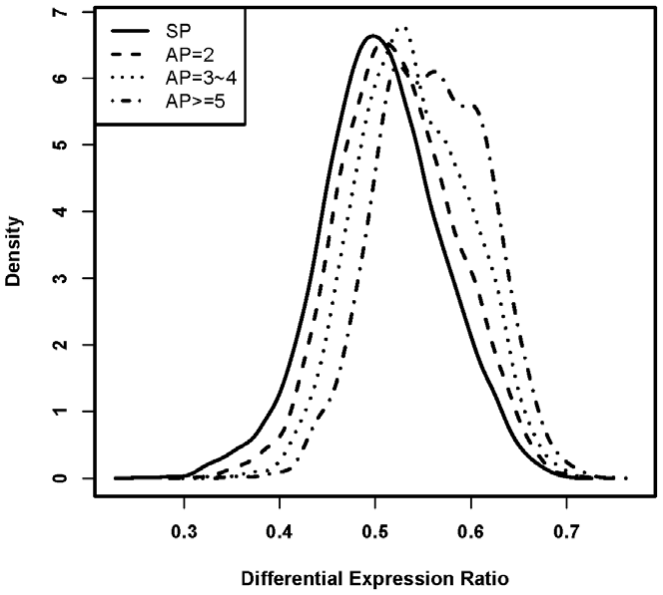
Distribution of differential expression ratio for each gene class. The figure (density plot) showed that genes with more alternative promoters are more likely to be differentially expressed. SP means gene with single promoter, while AP = 2, AP = 3∼4, and AP> = 5 means gene with only 2 promoters, 3 or 4 promoters, and at least 5 promoters, respectively.

For each human gene, we obtained its Differential Expression Ratio (DER) from the study by Chen *et al.* [16], [17]. The DER value of a gene is its frequency of differential expression in multiple microarray studies (see Methods section). As DER was derived from all available human microarray datasets deposited at GEO, it provided a comprehensive metric to measure the regulation diversity at expression level. To test the hypothesis whether genes with AP are more likely differentially expressed than genes with SP, we compared the DER between SP and AP genes. Of the genes with SP, the median DER was 0.50. In contrast, the genes with AP have median DER 0.53 (*P*<2.2e-16, Wilcox rank sum test). To test whether there is a general link between increasing number of promoter and differential expression among genes with AP, we divided the AP genes into three classes based on their number of promoters (AP = 2, AP = 3/4, AP> = 5, see methods). As shown in Figure 1, genes with more AP are more likely to be differentially expressed. The median DER was 0.52 for AP = 2 class (*P* = 2.2e-16, *vs.* SP), and increased to 0.54 for AP = 3/4 class (*P*<2.2e-16, *vs.* AP = 2 class). The median DER was further increased to 0.56 for AP> = 5 class (*P*<2.2e-16, comparing with that of AP = 3/4 class). Recent studies have shown that different tissues, cell types, developmental and/or disease stage are often regulated by distinct transcriptional factors, and there is considerable diversity in the composition of *cis*-regulatory elements in alternative promoters [2], [7], [18]. The increased number of alternative promoters from a single locus will provide increased flexibility and diversity of AP usage, and thereby generate either identical or distinct protein conducts in a tissues, cell lineage, stage, and time point specific manner. Such a diversifying and complex regulation control might contribute to the increased frequency of differential expression observed here for AP genes.

### AP Genes Are More Likely to Be Disease Susceptible

**Figure 2:**
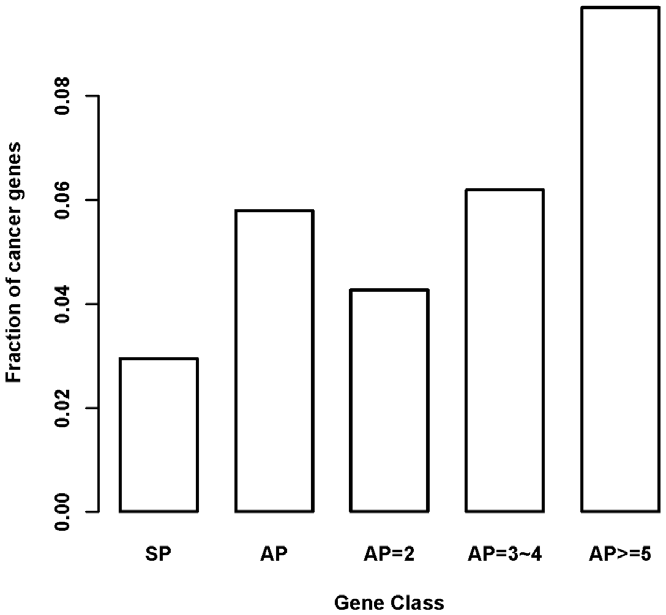
Fraction of cancer driver genes for each gene class. The figure showed that genes with more alternative promoters tend to be enriched with cancer driver gene. The Y-axis is the fraction of genes belonging to cancer driver gene in each gene class. SP means gene with single promoter while AP means gene with alternative promoters. AP = 2, AP = 3∼4, and AP> = 5 means gene with only 2 promoters, 3 or 4 promoters, and at least 5 promoters, respectively.

The study by Chen et al [17] has revealed that highly differentially expressed genes are more likely to be associated with disease. As we found that AP genes are more likely to be differentially expressed, it is expected that AP genes are more likely to be involved in disease. To confirm this positive link and quantify the extent to which a gene's promoter repertoire is associated with the likelihood of disease susceptibility, we first compiled a list of 775 human cancer genes which are likely to play casual roles in tumor formation and progression. We built a 2×2 contingency table using the number of cancer-driver gene and non-cancer-driver genes, and tested whether the fraction of cancer-driver genes is significantly increased from SP to AP gene classes using Fisher's exact test. As shown in Figure 2, the fraction of cancer-driver genes in SP class was 2.9%, and increased to 5.8% in AP class, an almost 2-fold increase (*P* = 2.2e-16). We further compare the fraction of cancer-driver genes between different AP classes. The fraction was found to be 4.3% for AP = 2 class (*P* = 0.00021, *vs.* SP), 6.2% for AP = 3/4 class (*P* = 0.00026, *vs.* AP = 2), and 9.7% for AP> = 5 class (*P* = 8.075e-05, *vs.* AP = 3/4).

**Figure 3:**
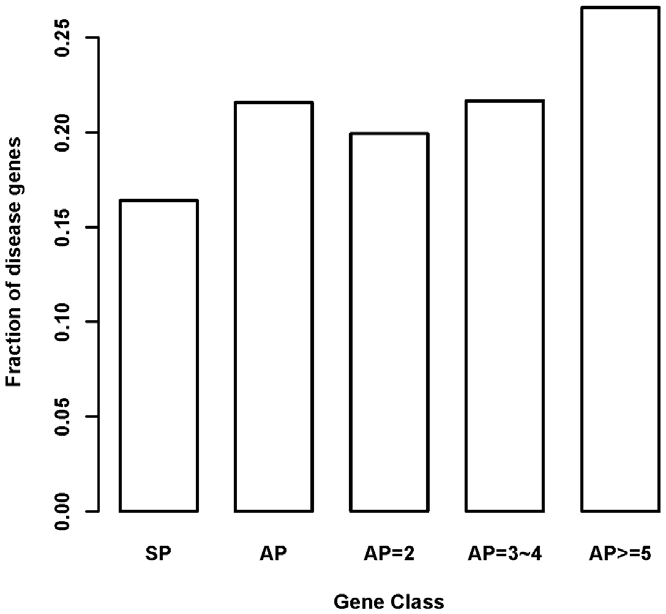
Fraction of disease genes for each gene class. The figure showed that genes with more alternative promoters tend to be enriched with disease gene. The Y-axis is the fraction of genes belonging to disease gene in each gene class. SP means gene with single promoter while AP means gene with alternative promoters. AP = 2, AP = 3∼4, and AP> = 5 means gene with only 2 promoters, 3 or 4 promoters, and at least 5 promoters, respectively.

In order to further characterize the general relationship between having increased promoter repertoire and the likelihood of being human disease susceptibility gene, we compiled a list of 3,392 curated human disease-associated genes. We again built 2×2 contingency tables and tested whether there is an increased fraction of disease gene from SP to AP gene classes using Fisher's exact test. As shown in Figure 3, the fraction of disease genes in SP class was 16.4%, and increased to 21.6% in AP class (*P* = 2.78e-16). The fraction was 19.9% for AP = 2 class (*P* = 2.497e-06, *vs.* SP), 21.7% for AP = 3/4 class (*P* = 0.04481, *vs.* AP = 2), and 26.6% for AP> = 5 class (*P* = 0.0004199, *vs.* AP = 3/4).

### AP Genes Are Longer in All Aspects of Gene Structure

**Figure 4:**
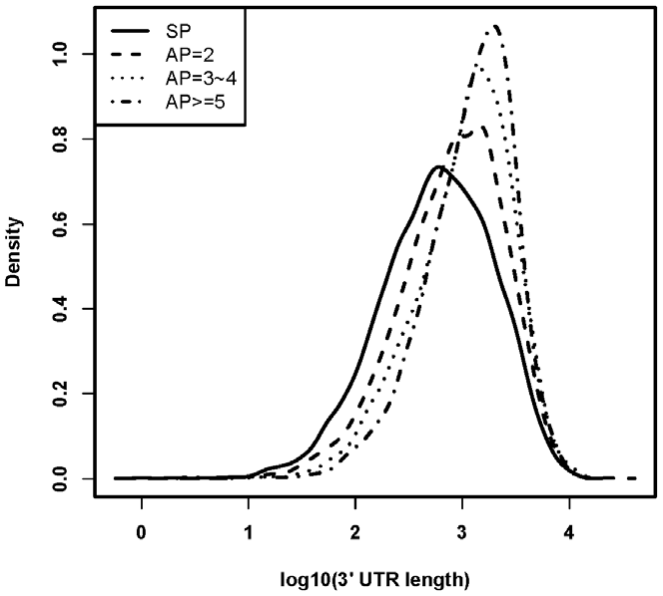
Length distribution for the 3′ un-translated region (3′ UTR) of each gene class. The figure (density plot) showed that genes with more alternative promoters tend to have longer 3′ UTR. SP means gene with single promoter, while AP = 2, AP = 3∼4, and AP> = 5 means gene with only 2 promoters, 3 or 4 promoters, and at least 5 promoters, respectively.

As shown in Table 1 and Figure S1, AP genes are significantly longer than SP genes in all aspects of the gene structure including genomic sequence, coding sequence (CDS), 5′ untranslated regions (5′ UTR), 3′ UTR, total exon, and total intron. AP genes also tend to have more exons and introns. Among AP genes, the class with more AP tends to be longer in all aspects of gene structure than the class with less AP (Table 1 and Figure S1). For example, the median of total intron length is 14.4, 25.2, 43.7 and 87.2 kb for SP, AP = 2, AP = 3∼4 and AP> = 5 gene class, respectively (*P*<2.2e-16, Wilcox rank sum test). As AP usage will lead to alternative usage of first exon, the increased number of AP will undoubtedly increase the degree of freedom for the extension of transcript region from the 5′ end [3]. However, it is remarkable that 3′ UTR, the region enriched for microRNA binding sites important for post-transcriptional regulation, also tend to be longer as the number of AP increases (Figure 4).

**Table 1:** Length parameter of each gene class.

	Genomic Sequence	CDS	5′ UTR	3′ UTR	Total Exon	Total Intron	# of Exon	# of Intron
**SP**	16,835 a	1,097	139	599	2,158	14,370	6 b	5
**AP**	41,017	1,638	183	1,062	3,274	37,314	11	10
***Pvalue*** c	<2.2e-16*	<2.2e-16	<2.2e-16	<2.2e-16	<2.2e-16	<2.2e-16	<2.2e-16	<2.2e-16
**AP = 2**	28,100	1,415	172	881	2,808	25,162	9	8
***Pvalue*** d	<2.2e-16	<2.2e-16	<2.2e-16	<2.2e-16	<2.2e-16	<2.2e-16	<2.2e-16	<2.2e-16
**AP = 3∼4**	48,058	1,763	188	1,178	3,492	43,650	12	11
***Pvalue*** e	<2.2e-16	<2.2e-16	2.153e-05	<2.2e-16	<2.2e-16	<2.2e-16	<2.2e-16	<2.2e-16
**AP> = 5**	90,787	2,296	207	1,325	4,178	87,204	17	16
***Pvalue*** f	<2.2e-16	<2.2e-16	0.000329	0.0005684	<2.2e-16	<2.2e-16	<2.2e-16	<2.2e-16

The table showed that genes with more alternative promoters tend to have increased length in all aspects of gene structure parameter. SP means gene with single promoter while AP means gene with alternative promoters. AP = 2, AP = 3∼4, and AP> = 5 means gene with only 2 promoters, 3 or 4 promoters, and at least 5 promoters, respectively.

a : Median length;

b : Median count;

c : Wilcoxon rank sum test, AP *vs.* SP.

d : Wilcoxon rank sum test, AP = 2 *vs.* SP.

e : Wilcoxon rank sum test, AP = 3∼4 *vs.* AP = 2.

f : Wilcoxon rank sum test, AP> = 5 *vs.* AP = 3∼4.

* : The Wilcoxon rank sum test function in R (wilcox.test) returns “*P*<2.2e-16” when *P* is smaller than 2.2e-16.

### AP Genes Are Associated with Increased Duplicability

**Figure 5:**
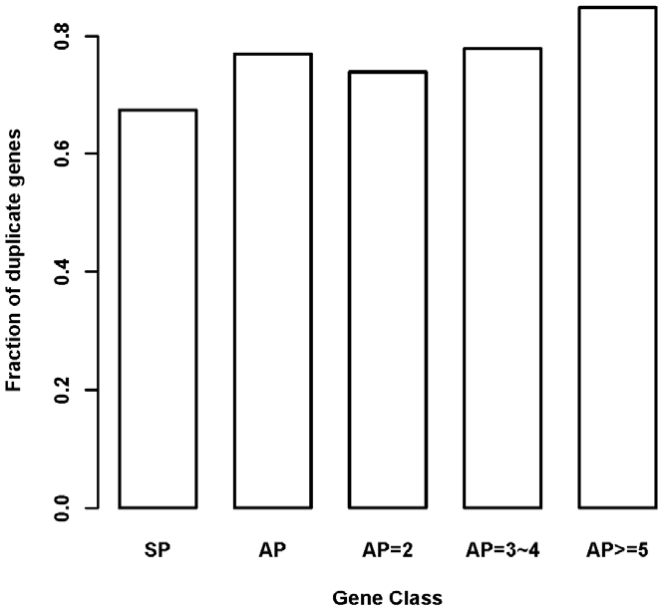
Fraction of duplicate genes for each gene class. The figure showed that genes with more alternative promoters tend to have increased duplicability. The Y-axis is the fraction of genes belonging to duplicate gene in each gene class. SP means gene with single promoter while AP means gene with alternative promoters. AP = 2, AP = 3∼4, and AP> = 5 means gene with only 2 promoters, 3 or 4 promoters, and at least 5 promoters, respectively.

We retrieved 14, 410 unique duplicate genes and 5, 226 unique singleton genes from Ensembl database via BioMart, with the fraction of duplicate gene about 73%. 10,665 of duplicate genes and 4,054 of singleton genes have curated promoter architecture from DBTSS (used in this work), with a similar ratio of duplicate gene (*i.e*, 72.5%). As shown in Figure 5, duplicate genes comprise 67% of SP genes, but make up 77% of AP genes (*P* = 1.087e-07, Fisher's exact test). The fraction was 74% for AP = 2 class (*P* = 0.002138, *vs.* SP), 78% for AP = 3/4 class (*P* = 0.08113, *vs.* AP = 2), and 85% for AP> = 5 class (*P* = 0.05049, *vs.* AP = 3/4).

### AP Genes Are More Likely to Be Associated with Hub

**Figure 6:**
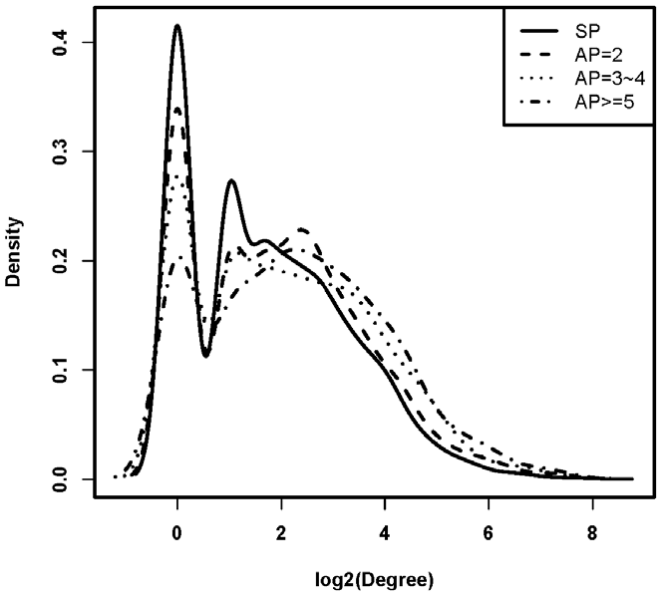
Distribution of node connectivity (degree) for each gene class in human protein-protein interaction network. The figure (density plot) showed that genes with more alternative promoters tend to have increased node connectivity. SP means gene with single promoter, while AP = 2, AP = 3∼4, and AP> = 5 means gene with only 2 promoters, 3 or 4 promoters, and at least 5 promoters, respectively.

We downloaded the manually curated human protein–protein interaction network from HPRD[19]. We found that the AP genes tend to have significantly more node connectivity (degree) than that of SP genes, and display a much stronger trend as the number of AP increases (Figure 6, *P< = *0.01, Wilcoxon rank sum test). The average connectivity of SP genes is 6.5, and increases to 10.5 for AP> = 5 gene class (*P<*2.2e-16).

#### Example of AP genes

To exemplify the characters of AP genes studied in this work, we described several genes whose alternative promoter usage has been shown in literatures. GNAS (guanine nucleotide binding protein, alpha stimulating activity polypeptide 1), is a G protein involved in hormonal regulation of adenylate cyclase. GNAS has ten potential alternative promoters supported by curated full-length c-DNA clones, and the switched recruitment of four of them has been found to generate multiple protein transcripts involved in metabolic regulation and development (For reviews, see Weinstein et al. [20] and Davuluri et al. [2]). GNAS has a high frequency of differential expression - differentially expressed in more than 69% of GEO dataset in which it was measured (DER value equals to 0.691). Promoter switching of GNAS has been found to plays a role in various diseases and tumorigenesis through loss of imprinting [21], [22], [23]. It is a disease gene of multiple syndromes including Albright hereditary osteodystrophy, pseudopseudohypoparathyroidism and McCune-Albright syndrome [24], [25]. It is a cancer driver gene of pituitary adenoma [26]. It is a duplicate gene, and the paralog is GNAL. The gene length of RUNX1 is 71.5 kb, well above the median of SP gene (16.8 kb). GNAS has 23 interacting partners in the protein-protein interaction network, comparing with an average connectivity of 6.5 for SP genes.

FGFR1 (fibroblast growth factor receptor 1), is a member of the fibroblast growth factor receptor family that binds to both acidic and basic fibroblast growth factors. FGFR1 has seven alternative promoters supported by curated full-length c-DNA clones, and at least of four of them have been shown to control the differential expression in a tissue- and cancer cell- specific manner [27], [28], [29], [30]. We found that FGFR1 is indeed frequently differentially expressed, with the DER value of 0.684. It is a disease gene of a number of syndromes including familial Pfeiffer syndrome [31]. It is cancer driver gene, implicated in the tumorigenesis of hematological malignancies including chronic myeloid leukemia, myeloid hyperplasia and non-Hodgkin's lymphoma [32]. It is a duplicate gene, with its paralogs including RET and FGFR2. FGFR1 has 18 exons and 5.9 kb exon length, comparing with the 6 exons and 2.2 kb exon length for SP gene. The protein-interaction network connectivity of FGFR1 is 36.

PDGFRA (platelet-derived growth factor receptor, alpha polypeptide), is a cell surface tyrosine kinase receptor for members of the platelet-derived growth factor family. The expression of PDGFRA is regulated by four potential alternative promoters, and the switched usage of two of them has been found to be involved in early human embryogenesis [33], [34]. The DER value of PDGFRA is 0.651, indicating that it is differentially expressed in more than 65% of GEO dataset in which it was measured. It is a key disease gene in hematologic disorder, involved in the gene fusions associated with the hypereosinophilic syndrome [35], [36]. It also serves as a well-documented cancer driver gene of gastrointestinal stromal tumor [37]. The paralog of PDGFRA, PDGFRB, has two alternative promoters and is also a cancer driver gene [38]. Compared with SP genes, PDGFA is both longer (69.1 kb) and connected by more interacting partners (24) in the protein-protein interaction network.

## Discussion

The functional role of alternative promoter usage in differential expression and/or disease susceptibility has been characterized for a bunch of genes. However, whether there is a positive link between a gene's AP repertoire and its likelihood of being differentially expressed and/or disease associated remains unknown. Based on a systematic analysis of promoter, microarray and disease gene in the public repositories, we found that compared with single-promoter genes, genes with alternative promoters are more likely to be differentially expressed and/or disease associated. Furthermore, our results showed that among AP genes, those with more promoters are more likely differentially expressed and/or disease susceptible.

Gene expression data has been frequently incorporated into the prioritization of disease candidate genes or SNPs. Recent translational study by Chen et al [17] has demonstrated that highly differentially expressed genes are more likely to have variants associated with disease, based on the analysis of all microarray data from GEO database. The finding that there is a positive association between differential expression and disease susceptibility marked a significant step towards the translation of gene expression data into disease gene prioritization. However, the molecular, genetic and genomic mechanism underlying this translation remains to be explored. Our study found that there is a general link between alternative promoter and differential expression and disease susceptibility. We further demonstrate that genes with increased number of alternative promoters are marked with features important to regulation complexity and disease origins, including increased gene length, duplicability and connectivity. While it remains to be explored for the positive prediction value of incorporating alternative promoter repertoire into disease gene prioritization, our results will be useful to understand the genomic mechanism underlying the translation from differential expression to disease susceptibility.

A better characterization of the role of alternative promoter usage on expression diversity and disease susceptibility requires a truly unbiased and comprehensive resource of alternative promoter activity, gene expression change and disease propensity. The DBTSS full-length cDNA derived alternative promoter data are taken from >160 distinct cDNA library of various cell types and tissue, and the GEO derived DER data are calculated based on 4,877 group-versus-group comparisons on 476 human GEO datasets. Although comprehensive, there is a possibility that both DBTSS and GEO data might be biased to certain biological niches. Thus, a future research direction will be to identify the separated effects in the analysis of alternative promoter *versus* differential expression, by classifying the different kinds of experiment in DBTSS and GEO (e.g., based on tissue, disease condition, and et. ac.). Also, it remains to be explored the effects of adopting alternative metric of differential expression and different definition of alternative promoters (e.g., varied cutoff of TSS clustering, other curated promoter database [39], and *et. ac.*). Similarly, the OMIM-based disease gene record is far from complete and historically biased to monogenic disorders. A more complete catalog of genes underlying different disease will alleviate the potential analysis bias to certain type of human disorders.

Recent technique developments in high-density promoter microarray and next-generation sequencing have enabled the genome-wide monitoring of alternative promoter activity and transcriptome change under different conditions [5], [6], [40], [41], [42]. Simultaneously, results from multiple genome wide association studies have shed light to the widespread involvement of regulatory variants including alternative promoters in disease association [43], [44], [45], [46]. By integrating the fast-accumulated data from these high-throughput studies and other functional genomics data, we expect that a more complete understanding of the mechanism of and extent to which alternative promoter usage has shaped human transcriptome and diseasome will be achieved.

In summary, based on a systematic analysis of promoter, microarray and disease gene in public repositories, we demonstrated that there exists a general link between a gene's alternative promoter repertoire and its expression diversity and/or disease susceptibility. Our further comparative analyses of AP vs. SP gene reveal several remarkable features of AP genes as a class. First, we found that AP genes tend to have longer length in all aspects of gene structure. As gene length is found to be positively related with the density of functional elements [47], it is reasonable to suggest that AP genes, with increased length in all aspects of gene parameter, subject to more sophisticated regulation besides transcriptional factor mediated promoter binding (e.g., alternative splicing [1], [48], microRNA mediated regulation [49], [50], [51], and *et. ac*.). Second, we showed that AP genes are associated with increased duplicability. Gene duplication has been widely appreciated as one of the factors underlying genetics variation, phenotypic diversity and disease mechanism [52]. Third, we observed that AP genes tend to have higher connectivity in protein-protein interaction network. The topological centrality of AP genes thus indicates that they play critical role in human physiological system [53]. Collectively, our analysis suggests that increasing AP repertories might be an important factor in shaping human genome, transcriptome and diseasome.

## Methods

We retrieved information of promoter annotation from DBTSS (Version 6.0, based on UCSC hg18) [54]. DBTSS determine alternative promoters using clustering of transcriptional start sites (TSS) by 500 bps, with TSS derived from collection (>160 distinct libraries) of experimentally determined 5′-end sequences of full-length cDNA clones. A total of 15,180 human RefSeq genes with curated full-length cDNA derived promoter architecture were obtained, which include 7,291 genes with Single Promoter (SP) and 7,889 genes with Alternative Promoter (AP). Among genes with AP, there are 3, 772 genes with two promoters (AP = 2), 2,941 with three or four (AP = 3∼4), and 1,176 with five or more (AP> = 5). The length parameter of gene structure was based on NCBI Reference Sequence (RefSeq) annotation. The 5′ UTR length is calculated from transcription start position and cording region start, while that of 3′ UTR from transcription end position and cording region end. For genes with multiple transcripts, the longest one is selected for length calculation.

We obtained the differential expression ratio (DER) of human genes from the study by Chen *et al.* [16], [17]. Briefly, the authors downloaded all curated human microarray-based gene expression datasets from the NCBI Gene Expression Omnibus (GEO) [55], [56], and conducted comprehensive group-versus-group comparisons within each dataset based on GEO annotated experimental variables (e.g., time, treatment, tissue, development stage *et ac.*) to identify differentially expressed (q value≤0.05, using SAM [57]) genes. For each human gene, the DER was calculated as the count of GEO datasets in which it was differentially expressed divided by the count of GEO datasets in which it was measured [17]. Only genes that were measured in at least 5% of all GEO datasets are included, which include 14,783 (97.4%) of the 15,180 genes with promoter annotations available from the DBTSS database.

We downloaded a manually curated collection of ∼380 human genes whose variants play a causal role in cancer (Cancer Gene Census database [14]). CGC is a regularly updated database to catalogue those genes for which mutations, deletions, and/or translocations have been causally implicated in cancer. We also compiled a set of ∼450 human cancer candidate genes, which are most likely to be key driver genes, based on recent large-scale sequencing of breast, colorectal, pancreatic and brain tumor genomes [15], [58], [59], [60]. The combination of these two datasets resulted in a list of 775 unique cancer driver genes.

We compiled a list of ∼2,380 known disease genes from the Morbid Map (MM) of the Online Mendelian Inheritance in Man (OMIM) [61]. Only the Morbid Map entries with the “(3)” tag, for which there is strong evidence that abnormality of the particular gene is causative to the disorder, were used to derive the list of human disease gene. We also downloaded a list of ∼2,360 human genes with annotated disease-associated variants from the latest Swiss-Prot database [62]. A combination of these two dataset resulted in 3,392 non-redundant human disease genes.

We used BioMart [63]to retrieve the complete set of human duplicate genes from EnsemblCompara GeneTrees database[64]. This corresponds to a total of 14,410 unique genes that have at least one duplicate copy in the human genome, and a total of 5,226 unique known singleton genes that have no duplicate copy.

We downloaded the manually curated human protein–protein interaction network from the Human Protein Reference Database [19], which is composed of 9,306 unique proteins and 35,023 protein–protein interactions (with self-interaction removed). The network degree was calculated using the NetworkAnalyzer plug-in [65] of Cytoscape package [66].

## Supporting Information

Figure S1 Length distribution for the gene structure parameter of each gene class. The figure (density plot) showed that genes with more alternative promoters tend to be longer in all aspects of gene structure. SP means gene with single promoter, while AP = 2, AP = 3∼4, and AP> = 5 means gene with only 2 promoters, 3 or 4 promoters, and at least 5 promoters, respectively.
(0.27 MB PDF)
