# Supplementary figures and images for: Increasing Alternative Promoter Repertories Is Positively Associated with Differential Expression and Disease Susceptibility
Source: PLoS One. 2010 Mar 1;5(3):e9482. doi: 10.1371/journal.pone.0009482 (PMC2830428; doi:10.1371/journal.pone.0009482)

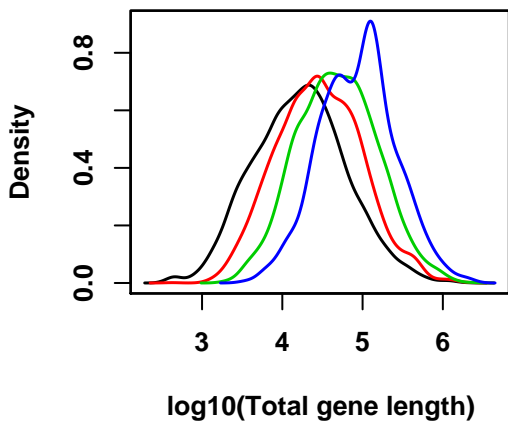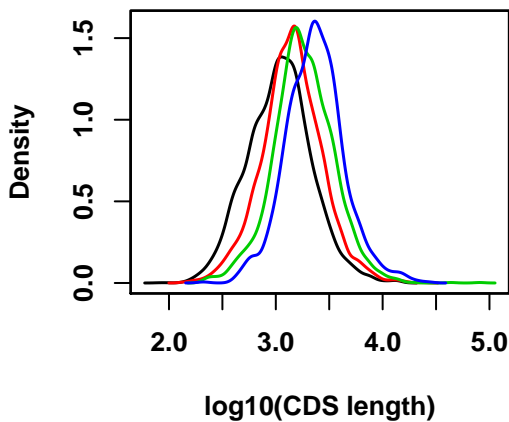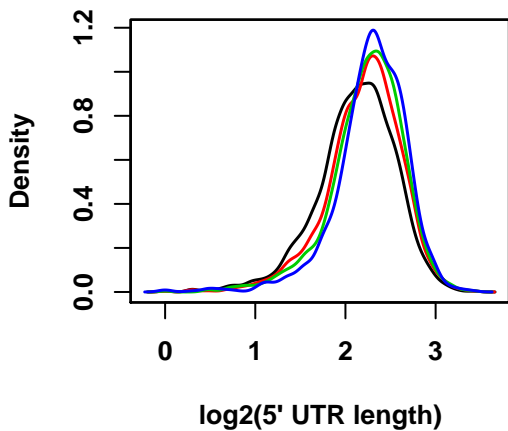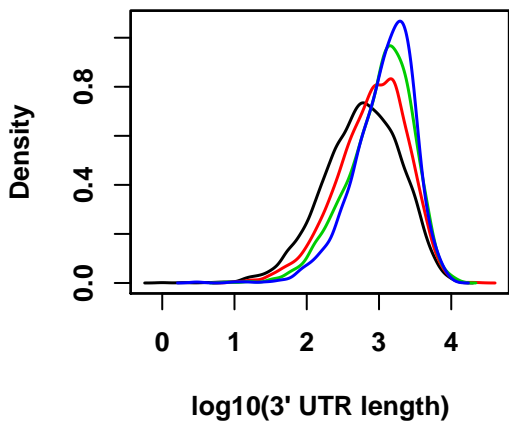

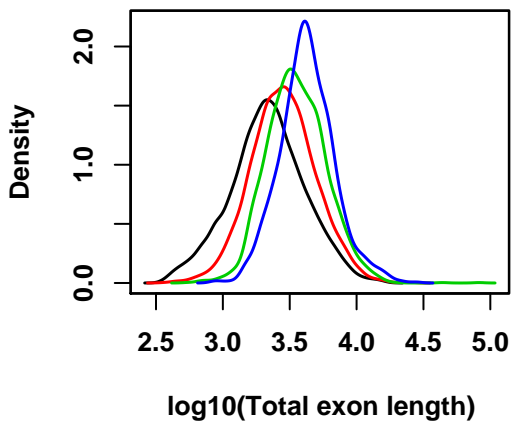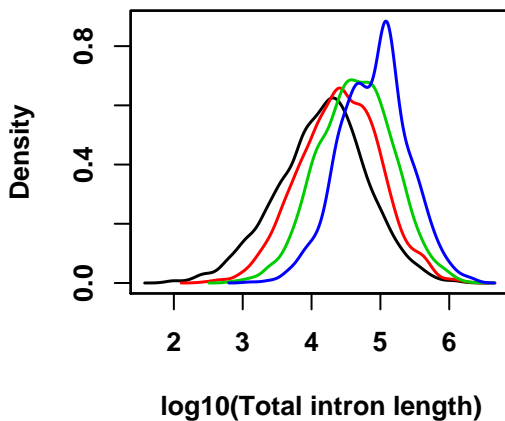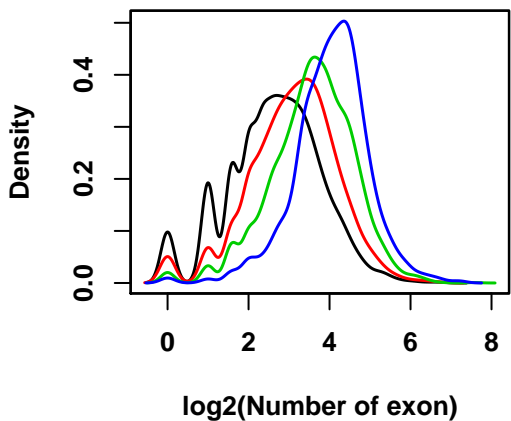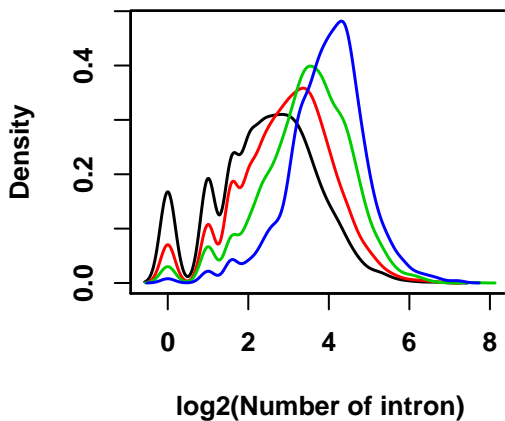

Supplement: Figure S1 — Length distribution for the gene structure parameter of each gene class. The figure (density plot) showed that genes with more alternative promoters tend to be longer in all aspects of gene structure. SP means gene with single promoter, while AP = 2, AP = 3∼4, and AP> = 5 means gene with only 2 promoters, 3 or 4 promoters, and at least 5 promoters, respectively. (0.27 MB PDF) [file pone.0009482.s001.pdf]
